# Tailoring of Seebeck coefficient with surface roughness effects in silicon sub-50-nm films

**DOI:** 10.1186/1556-276X-7-169

**Published:** 2012-03-05

**Authors:** Manoj Kumar, Anjana Bagga, S Neeleshwar

**Affiliations:** 1 Department of Physics, Indian Institute of Technology Delhi, Hauz Khas, New Delhi-110016, India; 2 University School of Basic and Applied Sciences, GGS Indraprastha University, Dwarka, New Delhi-110075, India

## Abstract

The effect of surface roughness on the Seebeck coefficient in the sub-50-nm scale silicon ultra thin films is investigated theoretically using nonequilibrium Green's function formalism. For systematic studies, the surface roughness is modelled by varying thickness periodically with square wave profile characterized by two parameters: amplitude (*A*_0_) and wavelength (*λ*). Since high Seebeck coefficient is obtained if the temperature difference between the ends of device produces higher currents and higher induced voltages, we investigate how the generated current and induced voltage is affected with increasing *A*_0 _and *λ*. The theoretical investigations show that pseudoperiodicity of the device structure gives rise to two effects: firstly the threshold energy at which the transmission of current starts is shifted towards higher energy sides and secondly transmission spectra of current possess pseudobands and pseudogaps. The width of the pseudobands and their occupancies determine the total generated current. It is found that current decreases with increasing *A*_0 _but shows a complicated trend with *λ*. The trends of threshold energy determine the trends of Seebeck voltage with roughness parameters. The increase in threshold energy makes the current flow in higher energy levels. Thus, the Seebeck voltage, i.e. voltage required to nullify this current, increases. Increase in Seebeck voltage results in increase in Seebeck coefficient. We find that threshold energy increases with increasing *A*_0 _and frequency (1/*λ*). Hence, Seebeck voltage and Seebeck coefficient increase vice versa. It is observed that Seebeck coefficient is tuneable with surface roughness parameters.

## 1. Introduction

**Figure 1:**
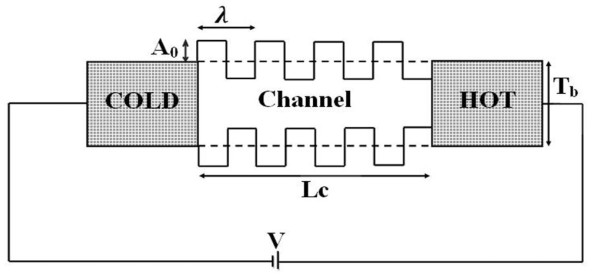
**The schematic diagram of simulated 2D Si structure**. Length of the channel is *L*_c_, average thickness is *T*_b _and roughness parameters are: amplitude (*A*_0_) & periodicity (λ). The bias is applied to nullify the current due to temperature gradient.

It is reported that when the two ends of the thermoelectric material are kept at different temperatures, the voltage is generated [1]. This effect is called as the Seebeck effect. This effect has useful applications as it shows that heat can be converted to electricity and vice versa. In the last few years due to quest for finding, the highly efficient ways to produce and use energy there has been an increased interest in the thermoelectric performance of a device [2]. Moreover, thermoelectric energy conversion paves the way for utilization of waste heat generated in the devices into useful electric power. The extraction of waste heat is an important issue, especially nowadays, when the devices are reaching the sub-50-nm scale. Thus, intensive research has been reported for the effect of dimensionality on the thermoelectric phenomenon [3-5]. As the dimensions reduce down to sub-50-nm scale, the surface roughness plays a crucial role in determining the performance of the device. In this scale, the spatial extent of variations due to roughness is comparable to the device dimensions [6]. Hence, the investigation on the effect of surface roughness is important and crucial [7-9]. In this article, we have made a systematic study of the surface roughness effects on Seebeck coefficient. The surface roughness has been modelled by varying the thickness of the film periodically with a square wave profile as shown in Figure 1. The roughness is characterized by two parameters: amplitude (*A*_0_) and wavelength (*λ*) of the square wave [10]. These parameters are analogous to the root mean square roughness and the roughness autocorrelation length which are commonly used to determine the surface roughness morphology [11]. We study the effect of roughness with increasing *A*_0 _and *λ *on the Seebeck effect. In other words, we investigate how does the induced voltage, *V*_Seebeck_, for a fixed temperature difference between the two ends of a device, is affected due to increasing *A*_0 _and *λ *of the surface roughness of the device. To clearly bring the role of surface roughness the electron transport is considered to be coherent inside the device. The phase coherent transport is possible in the devices with the dimensions in the sub-50-nm scale [12].

**Figure 2:**
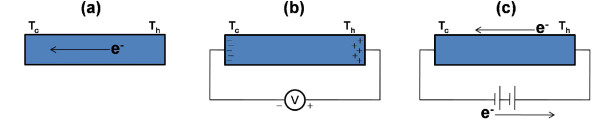
**Electron flow for different configurations of device**. **(a) **Electrons flow from hot end to cold end due to temperature gradient. **(b) **An induced emf is generated due to temperature gradient. **(c) **Voltage is applied to nullify the current due to temperature gradient. The voltage at which current becomes zero is called Seebeck voltage.

When the two ends of the device are subjected to a temperature difference, initially, there is a net flow of current *I*_net _from one end of the device to the other as shown in Figure 2a. This is so because, due to temperature difference, there is a different distribution of electrons in different energy states at the two ends. Hence, the number of electrons moving from the cold end to the hot end in an energy level '*ε*' is not the same as that of from hot end to cold end. This results in net flow of electrons in an energy level '*ε*'. The total current *I*_net _is obtained from adding the contributions from all the energy levels. This net flow of current results in an accumulation of electrons at one end which builds an electric field opposing the incoming electrons as shown in Figure 2b. Further accumulation of charge stops when no more electrons are able to make it due to the building of electric field. The voltage developed due to the electric field is called Seebeck voltage *V*_Seebeck_. Seebeck coefficient is obtained by dividing the Seebeck voltage by temperature difference [13]. As shown in Figure 2c, Seebeck voltage can be measured by calculating the amount of voltage required '*V*_applied_' to nullify the current '*I*_net_' due to temperature gradient. In this article, we focus on two important issues: firstly we investigate how does, for the same temperature difference between the two ends, *I*_net _changes with *A*_0 _and *λ*. Secondly, it has been observed that for some roughness parameters, though the *I*_net _decreases but the voltage applied to nullify it, *V*_Seebeck_, increases. We investigate the reasons behind this behaviour.

## 2. Theory

The expression for the current has been calculated using nonequilibrium Green's function formalism (NEGF) [14]. For the devices in the sub-50-nm scale quantum effects become important and quantum transport model is required for description of transport phenomenon. In this regards, NEGF formalism, in which quantum effects are inherent, provides an efficient framework to model the electron transport in thermoelectric devices. A brief description is given below and detailed development can be found in [15].

The isolated channel and its energy levels are described by Schrodinger equation using Hamiltonian H, potential U and energy eigenvalues of the electron *ε_α_*

(1) $$H\;\psi_{\alpha }(r)=\varepsilon_{\alpha }\psi_{\alpha }(r)$$

But when the channel gets connected to the contacts it becomes an open system and the electrons can come and go out of the channel. To represent such an open system, two extra terms get introduced in the Schrodinger equation. The Schrodinger equation becomes

(2) $$\left(EI-H-\sum_{1}-\sum_{2}\right)\left\{\psi \right\}=\left\{S\right\}$$

∑_1 _and ∑_2_, called self-energy, represent the coupling between the channel and the two contacts. ∑'*s *are a complex quantity. Its imaginary part signifies that the electrons can decay out of the channel.

(3) $$\text{Decay rate}\;=\frac{\Gamma }{\hbar }=\text{img}\left(\frac{\sum }{\hbar }\right)=\frac{i}{\hbar }\left(\sum -\sum^{+}\right)$$

The real part of ∑'*s *just lifts up the energy eigenvalues of the channel. The second term that gets introduced in the Schrodinger equation is {*S*}, it is called the source term and is like an actual source driving the system.

Solution of the Schrodinger equation is given by

$$\psi =\left[G\right]\left[S\right]$$

where

$$\left[G\right]={\left(EI-H-\sum_{1}-\sum_{2}\right)}^{-1}$$

(4) G=1EI−H−∑=1EI−H−Re(∑)−i Img(∑)

[*G*] is called the Green's function of the system. In the energy domain, Green function gives the energy eigenvalues for the eigenstates that are occupied in response to the applied impulse.

(5) $$\text{Density of states at energy}\;'E'=A\left(E\right)=i\left(G-G^{+}\right)=G\Gamma G^{+}$$

$$\text{As}\;\Gamma =\Gamma_{1}+\Gamma_{2}$$

$$A\left(E\right)=G\Gamma_{1}G^{+}+G\Gamma_{2}G^{+}$$

$$A\left(E\right)=A_{1}\left(E\right)+A_{2}\left(E\right)$$

*A*_1_(*E*) and *A*_2_(*E*) represent density of states due to coupling of the channel with contacts 1 and 2, respectively. The probability of filling of these states is determined by the Fermi Dirac distribution function, *f*^1^(*E*) and *f*^2^(*E*), of the contacts 1 and 2, which are at temperature *T *and *T+*Δ*T *respectively.

Carrier concentration is given by:

(6) $$\begin{array}{c} n\left(E\right)=A_{1}\left(E\right)f^{1}\left(E\right)+A_{2}\left(E\right)f^{2}\left(E\right)\\ =G^{n}\left(E\right) \end{array}$$

The net inflow of current in channel at contact 1 is the same as the net outflow of current at contact 2. Net current flow in channel at contact 1 is the difference between the inflow and outflow of current at contact 1.

$$\begin{array}{c} I\left(E\right)=\Gamma_{1}\left(E\right)f^{1}\left(E\right)A\left(E\right)-\Gamma_{1}\left(E\right)G^{n}\left(E\right)\\ =\Gamma_{1}\left(E\right)G\left(E\right)\Gamma_{2}\left(E\right)G^{+}\left(E\right)\left(f^{1}\left(E\right)-f^{2}\left(E\right)\right)\\ =T\left(E\right)\left(f^{1}\left(E\right)-f^{2}\left(E\right)\right) \end{array}$$

If *f*^1^(*E*) = 1 and *f*^2^(*E*) = 0 then *T*(*E*) represents the maximum current flow in the channel. For the electron transport in *x*-direction with confinement present in *z*-direction, the expression for I(E), after integrating over the energies along *y*-direction, becomes

(7) $$I\left(E_{x}\right)=T\left(E_{x}\right)\left(f_{1D}^{1}\left(E_{x}\right)-f_{1D}^{2}\left(E_{x}\right)\right)$$

(8) $$\text{Total current}=I=\underset{n_{z}}{\sum }\int I\left(E_{x}\right)dE_{x}$$

where *n_z _*is the subband index.

## 3. Results and discussion

**Figure 3:**
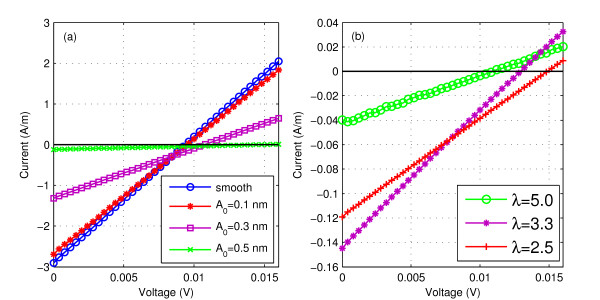
**Current-voltage characteristics for the 2D Si structure**. *L_c _*= 10 nm, *T_b _*= 2 nm, transport mass *m_x _*= 0.19 *m*_0_, quantization mass *m_z _*= 0.91 *m*_0 _and Δ*T *= 20 K. For (a) *λ *= 2.5 nm &*A*_0_'s = 0.1 nm, 0.3 nm, 0.5 nm and for (b) *A*_0 _= 0.5 nm &*λ*'s = 2.5 nm, 3.3 nm, 5 nm. Also in (a) *I*-*V *characteristics for smooth surface is shown for comparison.

The information about how does *I*_net_, i.e. the current due to the temperature gradient, and, *V*_Seebeck_, i.e. the voltage required to nullify it, depend on *A*_0 _and *λ*, is obtained by plotting the current voltage (*I*-*V*) characteristics for devices with different surface roughness parameters. Figure 3 gives the *I*-*V *characteristics for (a) different amplitudes: *A*_0_'s = 0.1 nm, 0.3 nm and 0.5 nm and *λ *= 2.5 nm and (b) different wavelengths: *λ*'s = 2.5 nm, 3.3 nm and 5 nm and *A*_0 _= 0.5 nm. The plot for smooth surface is given in Figure 3a for comparison. The temperature gradient of Δ*T *= 20 K is applied across the device. The graphs are obtained for the devices in the sub-50-nm scale by considering the parameters: length of the channel *L_c _*= 10 nm and average thickness *T_b _*= 2 nm. As *I*_net _is the current generated only due to temperature gradient with *V*_applied _= 0, it is given by the intercept with the *y*-axis. It is observed from Figure 3a, b that *I*_net _decreases with increasing *A*_0 _but with *λ *there is no definite trend. *V*_Seebeck _is the voltage applied to make the current due to temperature gradient equal to zero and is obtained by the intercept with the *x*-axis. It is observed from Figure 3a, b that *V*_Seebeck _increases with increasing *A*_0 _and 1/*λ*.

The above trends of *I*_net _and *V*_Seebeck _with the roughness *A*_0 _and 1/*λ *can be understood by examining the current flowing through each energy level *I*(*E*). As seen from Equation 7, *I*(*E*) is given as the product of transmission *T*(*E*) and $\left(f_{1D}^{\text{cold}}\left(E\right)-f_{1D}^{\text{hot}}\left(E\right)\right)$, i.e. difference in the occupancy of cold $f_{1D}^{\text{cold}}\left(E\right)$ and hot junctions $f_{1D}^{\text{hot}}\left(E\right)$. The transmission *T*(*E*) gives the maximum current through the energy level '*E*' and depends on the density of states *A*(*E*) and velocity, i.e. rate of flow of electrons in and out of that energy level. As such the geometry of the device does not affect the occupancy and hence $\left(f_{1D}^{\text{cold}}\left(E\right)-f_{1D}^{\text{hot}}\left(E\right)\right)$ but it affects the transmission *T*(*E*) of electrons from energy level *E *through density of states *A*(*E*). Thus, the trends of *I*_net _and *V*_Seebeck _with *A*_0 _and *λ *can be understood by examining how the transmission gets affected by roughness.

### 3.1. Effect of roughness on transmission spectra

**Figure 4:**
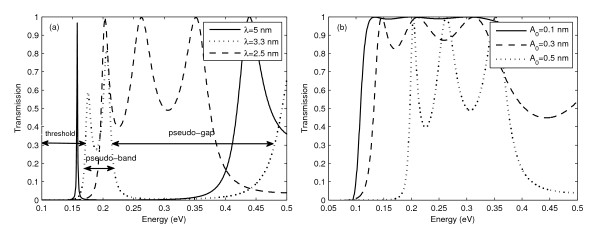
**Energy resolved transmission spectra at zero applied voltage**. For (**a) ***A*_0 _= 0.5 nm &*λ*'s = 2.5 nm, 3.3 nm, 5 nm and for (**b) ***λ *= 2.5 nm &*A*_0_'s = 0.1 nm, 0.3 nm, 0.5 nm. Temperature gradient Δ*T *= 20 K for both (**a**, **b)**.

Figure 4 gives the plots of *T*(*E*) for (a) roughness wavelength: *λ*'s = 2.5 nm, 3.3 nm and 5 nm with *A*_0 _= 0.5 nm and (b) roughness amplitude: *A*_0_'s = 0.1 nm, 0.3 nm and 0.5 nm with *λ *= 2.5 nm. The following trends are observed: (i) threshold energy, i.e. the 'onset' energy at which the transmission starts, increases with increasing *A*_0 _and 1*/λ*. (ii) There are regions of psuedobands and pseudogaps. The width of the pseudobands decreases with increasing *A*_0 _and *λ*. Within the pseudobands, there are peaks in transmission at some energy. The phenomenon of increase in threshold energy has also been observed experimentally in quantum wells with thickness < 4 nm [16]. We would like to mention here that it has been observed only for thicknesses below 4 nm because surface roughness effects become prominent for ultra thin films. The criterion for the thickness below which surface roughness effects become prominent is discussed in [16]. So, our discussions can be experimentally validated only for ultra thin films. We will analyse the implications of the above trends on *I*_net _and *V*_Seebeck _but before that we discuss in brief the presence of the above trends.

**Figure 5:**
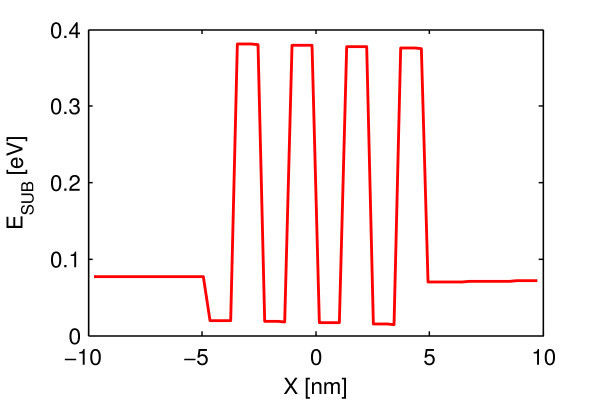
**Suband energy profile along the length of the device**. Roughness amplitude (*A*_0_) = 0.5 nm and periodicity (*λ*) = 2.5 nm. The regions of channel at which confinement is high, subbands energies are high and vice versa.

The increase in threshold energy occurs due to the varying thickness seen by the electron while crossing the channel. The regions of the channel where the thickness is large, the confinement is small and the regions where the thickness is small, the confinement is high as seen from Figure 1. Since the lowest value of *ε_z_*, which corresponds to the subband energy, depends on the confinement it alternates with the same periodicity as the thickness as shown in Figure 5. The value of *ε_z_*, i.e. subband energy is large for high confinement and vice versa. The subband energy corresponds to the potential energy seen by the electron. The electron crossing the channel experiences this varying potential energy landscape which was not the case when the surface was smooth. As the electron crossing the channel moves from the region of low potential energy to the high potential energy it feels an additional confinement effect which was not present for smooth surface. This additional confinement effect causes the increase in lowest energy which the electron can take. Thus, the threshold energy, which corresponds to the lowest energy eigenvalue, increases. The confinement effect will be more for increasing *A*_0 _as the electron while moving from low potential energy to high potential energy region sees higher barrier heights. Thus, the lowest energy eigenvalue, i.e. threshold energy, increases with increasing *A*_0_. The increase in threshold energy with increasing 1/*λ *can be explained on the similar lines. The longer *λ *with the same *A*_0 _corresponds to the wider regions of the low potential energy with the same barrier height. This means the confinement effect reduces with increasing *λ*. Hence, the threshold energy decreases with increasing *λ*. In other words threshold energy increases with increasing 1/*λ*.

The presence of pseudobands and pseudogaps, a feature of only rough surfaces, is also due to alternate regions of low and high potential energy seen by the electron while crossing the channel. The energy eigenvalues for an electron in such a periodic potential energy profile are given by Kronig Penny model. The solution of the Schrodinger equation for such a potential energy profile shows the presence of energy bands and energy gaps. The investigations on the bandwidth (BW) of the pseudobands with respect to roughness parameters is important as the width of the pseudoband determines the area under the transmission curve and play an important role in determining the total current *I*_net_. It is seen from Figure 4a, b that BW of the pseudobands decreases with increasing *A*_0 _and *λ*. These trends of BW on roughness parameters can be understood by looking, again, at the Kronig Penny model. According to Kronig Penny model, if the barrier heights and widths of the periodic potential profile are high, then the BW's are small. This is so because higher barrier heights and widths give smaller tunnelling probability. As the tunnelling probability becomes smaller the BW reduces. Along the same lines increasing *A*_0_, which corresponds to higher barrier heights, and increasing *λ*, which corresponds to larger barrier widths of the pseudoperiodic potential profile, would give rise to smaller BW's. Thus BW decreases with increasing *A*_0 _and *λ*.

### 3.2. Effect of roughness on total current *I*_net_

As *I*_net _represents the current only due to temperature gradient with *V*_applied _= 0, it is given by the intercept with the *y*-axis of *I*-*V *characteristics. It is observed from Figure 3a that *I*_net _decreases with increasing amplitude but Figure 3b shows that there is no definite trend with *λ*. These features can be explained by considering that total current depends on the bandwidth of the pseudobands and their occupancy. For increasing *A*_0 _as seen from Figure 4b, threshold energy increases and BW of the pseudobands decreases. The smaller bandwidths along with their presence at higher energy sides result in the decrease in occupancy of these bands. This, in turn, decreases the total no. of carriers contributing to the current. Thus, *I*_net _decreases with increasing *A*_0_. The trends of *I*_net _with *λ *are complicated. As seen from Figure 4a though the pseudobands are present at lower energy sides with increasing *λ *but their BW decreases. Thus, on one hand their presence at lower energy sides increases the occupancy on the other hand smaller bandwidths decrease the total occupancy which is obtained by summing the occupancy from all energy levels. These two competing features complicate the trends of *I*_net _with *λ*. Figure 4a shows that BW for *λ *= 3.3 nm is smaller than *λ *= 2.5 nm but since the band is present at lower energy sides its occupancy is more. This results in more current for *λ *= 3.3 nm than for *λ *= 2.5 nm. For *λ *= 5 nm the BW is so small that the current is the least.

### 3.3. Effect of roughness on Seebeck voltage *V*_Seebeck _and Seebeck coefficient

**Figure 6:**
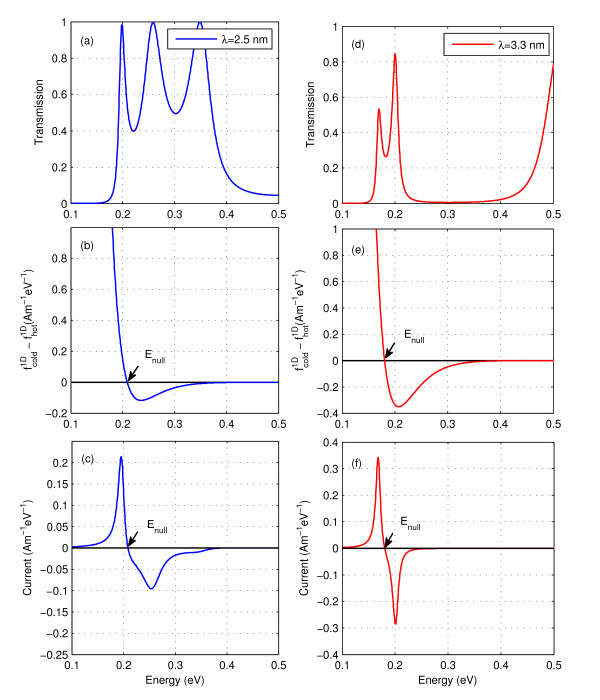
**Energy resolved transmission, Fermi Dirac occupancy and current for *λ *= 2.5 nm and 3.3 nm with *A_0 _*= 0.5 nm**. Energy resolved transmission is shown in **(a, d)**, difference in the Fermi Dirac occupancy of cold and hot ends is shown in **(b, e) **and current is shown in **(c, f)**. For **(a-c) ***λ *= 2.5 nm and for **(d-f) ***λ *= 3.3 nm. *E*_null _shifts towards higher energy sides with decreasing wavelength.

As already mentioned *V*_Seebeck _increases with increasing *A*_0 _and 1/*λ*. This trend can be understood by examining the circumstances for which *I*_total _(= *I*_net_) becomes zero. *I*_total _is the sum of current from all energy levels, i.e. *I *= ∫*I*(*E*)*dE*. Figure 6c gives *I*(*E*) versus *E *for *λ *= 2.5 nm and *A_0 _*= 0.5 nm. It is seen that below *E*_null _the current is +ve and above *E*_null _the current is -ve. Hence, the total current obtained by the sum of current from all energy levels is small. We need to check when does it become zero. As seen from Equation (7), *I*(*E*) is product of transmission *T*(*E*) and $\left(f_{1D}^{\text{cold}}-f_{1D}^{\text{hot}}\right)$, i.e. difference in the occupancy of cold $f_{1D}^{\text{cold}}$ and hot junctions $f_{1D}^{\text{hot}}$. Since *T*(*E*) ≥ 0 for all energies therefore current changes sign whenever $\left(f_{1D}^{\text{cold}}-f_{1D}^{\text{hot}}\right)$ changes sign as seen from Figure 6b. For those energy levels in which the number of electrons at the cold side are more than the number of electrons at the hot side, the net current flows from cold to hot and $\left(f_{1D}^{\text{cold}}-f_{1D}^{\text{hot}}\right)$ is +ve and *I*(*E*) is +ve. These are the energy levels below *E*_null_. It is vice versa for the energy levels above *E*_null_. At *E*_null_, $\left(f_{1D}^{\text{cold}}-f_{1D}^{\text{hot}}\right)$ = 0. This *E*_null _is a function of *V*_applied _as occupancies of energy states at cold side, $f_{1D}^{\text{cold}}$ and hot side, $f_{1D}^{\text{hot}}$, depend on *V*_applied_. *E*_null _shifts towards higher energy sides with increasing *V*_applied_. We apply voltage to shift *E*_null _to such an extent that total current coming from energy levels below *E*_null _balances the total current coming from energy levels above *E*_null _so that the total current becomes zero.

To understand how does *E*_null _shifts towards higher energy states with increasing *V*_applied_, we observe that as the threshold energy increases the electrons occupy higher energy states and the current contributions come from higher energy levels. The net current *I*_net_, which flows from hot side to cold side is also made of electrons flowing in higher energy levels. We need to apply voltage, *V*_Seebeck_, to nullify this current *I*_net_. Thus, we need to increase the potential of the cold side so that electrons at the cold side occupy higher energy states to balance this current. Thus, as the threshold energy increases we need to apply higher *V*_Seebeck _to balance the net current. Also, since the occupancy of higher energy states at the cold side increases with increasing *V*_Seebeck _the *E*_null_, for which $\left(f_{1D}^{\text{cold}}-f_{1D}^{\text{hot}}\right)=0$, shifts towards higher energy sides. Since the threshold energy increases with increasing *A*_0 _and 1/*λ*, hence Seebeck voltage *V*_Seebeck _increases with the increasing roughness amplitude and frequency. Since Seebeck coefficient is obtained by taking the ratio of *V*_Seebeck _with temperature difference Δ*T*, i.e. *S *= *V*_seeback_/Δ*T*, therefore the increase of *V*_Seebeck _for fixed Δ*T *causes the increase in Seebeck coefficient with the similar trends. It is seen from Tables 1 and 2 that Seebeck coefficient increases with increasing *A*_0 _and 1/*λ*. The dependence of Seebeck coefficient on roughness parameters suggest that it can be tailored by choosing the appropriate roughness parameters.

**Table 1:** Variation of Seebeck coefficient for different roughness amplitudes

*A*_0 _(nm)	*S *(μV/K)
Smooth	470
0.1	475
0.3	537
0.5	740

Roughness wavelength (*λ*) = 2.5 nm is fixed.

**Table 2:** Variation of Seebeck coefficient for different roughness wavelengths

λ (nm)	S(μV/K)
Smooth	470
5.0	545
3.3	646
2.5	740

Roughness amplitude (*A*_0_) = 0.5 nm is fixed

For the devices if *A*_0 _is increased at constant *λ *it is observed that though *I*_net _decreases but still *V*_Seebeck _increases. This happens because BW decreases and threshold energy increases. Smaller BW's at higher energy sides reduce occupancy and hence *I*_net_. Though the current reduces, it is made up of electrons present in higher energy levels and as already explained higher voltages are required to nullify the current from higher energy states.

The discussions show that it is the increase in threshold energy which causes the current to flow in the higher energy levels and hence results in increase in Seebeck coefficient. This result implies that any physical geometry whether periodic or aperiodic which results in an increase in threshold energy will show an increase in Seebeck coefficient. It also implies that if the threshold energy dependence on roughness parameters become feeble then Seebeck coefficient will saturate, i.e. it will not change much with the change in roughness parameters. The detailed investigations on the above two implications are in progress.

## 4. Conclusions

The surface roughness causes the net current *I*_net_, produced due to the temperature gradient, to decrease with increasing roughness *A*_0 _but trends with *λ *are complicated. The voltage *V*_Seebeck_, required to nullify this *I*_net _shows a definite trend. It increases with increasing *A*_0 _and 1/*λ*. Increase in *V*_Seebeck _results in increase in Seebeck coefficient. The reasons for the above trends have been attributed to the varying periodic thickness seen by the electron crossing the channel. The periodically varying thickness corresponds to varying confinement and hence periodically varying subband energy. The subband energy corresponds to the potential energy seen by the electron. For the electron moving in a pseudoperiodic potential, the transmission spectra shows the following features: (1) The threshold energy at which the transmission of current starts is shifted towards higher energy sides. It increases with increasing *A*_0 _and 1/*λ*. (2) There are regions of pseudobands and pseudogaps. The BW of the pseudobands decreases with increasing *A*_0 _and *λ*.

The above features of transmission spectra result in following trends for *I*_net _and *V*_Seebeck_: (1) It is observed that BW decreases and shift towards the higher energy sides for increasing *A*_0_. Both these features result in reduced occupancy of the band and hence total carrier concentration. Since *I*_net _depends on the total carrier concentration thus *I*_net _reduces with increasing *A*_0_. (2) It is observed that pseudobands are present at lower energy sides with increasing *λ *but their BW decreases. Thus, on one hand the presence of bands on the lower energy side increases the occupancy and on the other hand the smaller bandwidth reduces the total occupancy. These two competing features give rise to complicated trends of *I*_net _with *λ*. (3) The trends of threshold energy determine the trends of *V*_Seebeck _with roughness parameters. The increase in threshold energy results in the electrons to occupy higher energy states. Thus the net current *I*_net _flowing from hot to cold end is made up from higher energy levels. To nullify this current, the occupancy of higher energy states at the cold end is increased by applying higher voltages. Thus higher threshold energy requires higher applied voltages, *V*_Seebeck_, to nullify the current. Since threshold energy increases with increasing *A*_0 _and 1/*λ*, hence *V*_Seebeck _and Seebeck coefficient increases vice versa. (4) The dependence of Seebeck coefficient on roughness parameters suggest that it can be tailored by choosing the appropriate roughness parameters.

## Competing interests

The authors declare that they have no competing interests.

## Authors' contributions

MK carried out the NEGF calculations and implemented the code. AB and SN conceived of the study. AB participated in the NEGF calculations and implementation of the code. SN, with the perspective of designing the device in his lab, participated in the discussions of theoretical results and provided the information of the related experimental works. All authors read and approved the final manuscript.
